# Prevalence of Keel Bone Damage in Red Jungle Fowls (*Gallus gallus*)—A Pilot Study

**DOI:** 10.3390/ani10091655

**Published:** 2020-09-15

**Authors:** Käthe Elise Kittelsen, Per Jensen, Jens Peter Christensen, Ingrid Toftaker, Randi Oppermann Moe, Guro Vasdal

**Affiliations:** 1 Animalia—The Norwegian Meat and Poultry Research Centre, Lorenveien 38, NO-0585 Oslo, Norway; guro.vasdal@animalia.no; 2 AVIAN Behavioural Genomics and Physiology Group, IFM Biology, Linkoping University, 58183 Linkoping, Sweden; per.jensen@liu.se; 3 Department of Veterinary & Animal Sciences, University of Copenhagen, 1165 Copenhagen, Denmark; jpch@sund.ku.dk; 4 Faculty of Veterinary Medicine, NMBU—Norwegian University of Life Sciences, PO Box 8146 dep., NO-0033 Oslo, Norway; ingrid.toftaker@nmbu.no (I.T.); Randi.moe@nmbu.no (R.O.M.)

**Keywords:** red jungle fowl, keel bone fracture, keel bone damage, laying hen, animal welfare, poultry welfare

## Abstract

**Simple Summary:**

The keel bone of laying hens is prone to deviations and fractures. Recent studies in the last decade report high prevalence of this welfare concern. The causative factors are not clear; however, selection for efficient egg production has been suggested as a major contributing factor. An important step to shed light on the role of selective breeding as an underlying cause of keel bone damage (KBD) in modern laying hens is to evaluate the keel bones of the ancestor, the red jungle fowl. The aim of this study was therefore to describe the prevalence of KBD in a study group of 29 red jungle hens and roosters by autopsy. No fractures were detected in the roosters, one had a very slight deviation. One of the hens had a fracture to the keel and 10 hens had a very slight deviation. Large scale studies are needed to disentangle the effect of different factors on keel bone damage.

**Abstract:**

Keel bone damage (KBD) is a highly prevalent problem in commercial egg production. KBD consists of two different conditions affecting the keel: Keel bone deviation and keel bone fractures (KBF). Deviations are linked to pressure on the keel, e.g., from perching. The causative factors for KBF are not clear; however, selection for efficient egg production has been suggested as a major contributing factor. An important step to shed light on the role of selective breeding as an underlying cause of KBF in modern laying hens is to evaluate the keel bones of the ancestor, the red jungle fowl. To the authors’ knowledge, this has never previously been published. The aim of this study was therefore to describe the prevalence of KBD in a study group of red jungle hens and roosters housed in an aviary system. The present study examined 29 red jungle fowls 112 weeks of age post-mortem; 12 hens and 17 roosters. Keel bones were evaluated by external palpation for deviations and fractures. Palpation was followed by autopsy. No fractures were detected in the 17 roosters; one had a very slight deviation. Of the 12 red jungle hens in this pilot study, one had a single fracture and 10 hens had a very slight deviation.

## 1. Introduction

The term keel bone damage (KBD) consists of two conditions affecting the keel bone: keel bone deviations and keel bone fractures (KBF). Keel bone deviations are defined as a bone with an abnormally shaped structure not resulting from a fracture, while keel bone fractures are defined as sharp bends or fragmented sections of the keel bone [1]. Proportions of hens with keel bone damage ranging from 30 to 97% [2,3,4] have been reported across different production systems for commercial laying hens [5,6,7]. Keel bone fractures may be associated with pain [8,9], reduced mobility, altered behavior, and altered affective state [9,10,11,12,13]. The high prevalence combined with the welfare implications for the hens makes keel bone damage one of the most urgent welfare problems faced by laying hen industry today [6,14].

Comparing results from different studies of KBD occurrence or associated risk factors is difficult and must be done with caution, mainly for two reasons: 1. It is not always clear if the study has been investigating keel bone fractures or keel bone deviations; and 2. the assessment method varies between studies. The most common method to assess keel bones is external palpation [1,15]. A callus is formed during fracture healing and can be detected as uneven structures on the lateral surface of the keel bone [1,6]. However, as the callus takes some time to develop, new fractures without callus formation may be missed during palpation [16]. Furthermore, fracture sites in motion will create more periosteal callus formation than fracture sites with less motion [17]. Thus, the evaluation of KBF by palpation only might underestimate the occurrence, especially in caged hens, compared to loose housed birds with more activity and more callus [18]. In addition, the accuracy of palpation depends on prior experience of the person performing the examination; underestimation is a well-known problem [1,2,15,19]. In addition, the majority of keel bone fractures are located at the caudal tip of the keel bone, and on the dorsal side of the bone [18], which is inaccessible for palpation. The gold standard diagnostic methods are therefore considered to be x-ray or autopsy [15,16,19]. These methods are sensitive enough to detect fresh fractures without calluses and fractures on the dorsal side of the keel bone [1,16].

The etiology of KBF and deviations are assumed to differ [7]. Deviations are linked to pressure on the keel from perching [6]. The causative factors for KBF are not as clear [20]. Different explanations have been suggested: Trauma due to collisions within the housing system [21], genetic susceptibility [22,23], selection for high egg production [23], bone fragility [24], early onset of lay [4], and late ossification of the caudal part of the keel making it vulnerable during egg production [18]. Several of these factors are linked to selective breeding for efficient egg production. The wild ancestor of modern layers lays around four to six eggs per breeding season [25]. Around the year 1900, a selected egg-laying hen produced 83 eggs yearly [26]. Today, modern layers produce close to 330 eggs per hen per year. The association between egg production and keel bone fracture is supported by the absence of fractures in males [27]. Further evidence of a link between egg production and fractures was gained in a controlled trial where egg production was suppressed completely by deslorelin acetate, resulting in normal, fracture free keel bones [28].

The modern laying hen (*Gallus gallus*) descends from the red jungle fowl, which still lives in its wild form in South East Asia today. If selection for efficient egg production is an underlying cause for KBF in the modern laying hen, we hypothesize that the occurrence of KBF will be lower in the red jungle fowl as found in the modern hybrids. Therefore, the aim of this pilot study was to describe the prevalence of keel bone fractures and deviations in a study group of semi-selected red jungle hens and roosters housed in an aviary system. To ensure sufficient diagnostic accuracy, the study used both palpation and autopsy to avoid underestimation or uncertain results.

## 2. Material and Method

### 2.1. Birds and Management

The study was approved by the Linköping council for ethical licensing of animal experiments, ethical permit no. 14916-2018.

The study group consisted of 29 jungle fowl; 12 males and 17 females, kept at the University of Linköping. The birds were not purebred jungle fowls; they originated from two different zoo populations originally crossed to increase genetic variability. The two crossed populations were from Copenhagen zoo and Götala research station (SLU) [29]. The birds were housed in a modified multitier aviary system, divided into pens of 3 × 3 × 3 m (Vencomatic Group, Eersel, and the Netherlands). Each pen housed 30 birds. This equals a stocking density lower than EU regulations [30]. In addition, all birds had unlimited access to a 3 × 3 × 2 m outdoor area. The aviary, and as such all pens, consisted of two tiers; the first was 100 cm above the floor and the second tier 220 cm above the floor. The first tier had metal wired floor. The floor level was covered with wood chips as dustbathing material. All levels were equipped with perches of metal. Nest boxes were situated in the first tier. The nipple drinkers were located on the first tier and on the floor level. The automatic feeding chains in the first tier provided the birds with commercial ad libitum feed (supplier: Lantmännen AB, 104 25 Stockholm). We selected two pens for keel bone examination; these pens contained 29 birds at the time of culling. The birds were culled by cervical dislocation and decapitation at the age of 112 weeks.

### 2.2. Production Data

Onset of lay was 23 weeks. The average laying performance in the red jungle fowls in the source population was 70% during peak production (April to August). During the winter months the egg production dropped to 40–50% and 0% during molting and brooding. Average egg weight during the production period was 23 g (SD 19.8). Body weight at 200 days for the hens was on average 799.5 g (SD 130.1) and for the roosters on average 1119.1 g (SD 136.3).

### 2.3. Keel Bone Examination

The birds were shipped frozen, express overnight, from the University of Linköping, Sweden to Oslo, Norway for examination. Evaluation of the keel bones were performed by an experienced poultry veterinarian, trained by the EU Cost Keel Bone Action’s training school. A deviation was defined as a bone with an abnormal shape, not associated with fracture. Deviations were diagnosed visually and by palpation and scored on a binary scale: Yes or no, in accordance with the Simplified Keel Assessment Protocol (SKAP) [1].

A keel bone was classified as a having a fracture if there was visible or palpable callus or a thin, dark, slightly protruding line on the dorsal side, which is a fracture without callus formation. The keel bone was first palpated for factures on a binary SKAP-scale: Yes or no [1]. External keel bone palpation was followed up by autopsy, including inspection of the dorsal side of the keel bone before giving the binary score. Additionally, during autopsy, fractures were classified according to which part of the keel the fracture was located; cranial, middle, or caudal part, along with the total number of fractures per bird.

### 2.4. Statistics

Proportions with confidence intervals were calculated in Stata [31].

## 3. Results

### 3.1. Keel Bone Deviations

A total of 11 of the 29 keel bones presented a deviation: One male and 10 females, see Table 1. All deviations were very slight and could be palpated as a mild bending in the bone. The deviations had similar locations; in the cranial part of the middle third section of the keel bone.

### 3.2. Keel Bone Fractures

**Figure 1:**
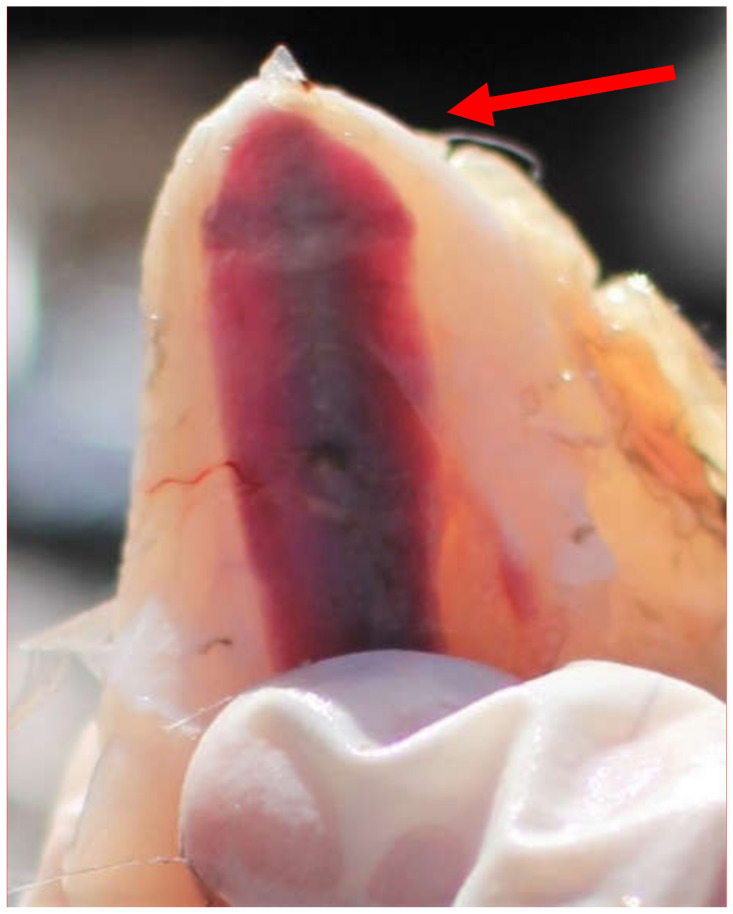
A keel bone with a dark fracture line and slight callus formation (indicated with a red arrow), caudo-dorsally.

**Figure 2:**
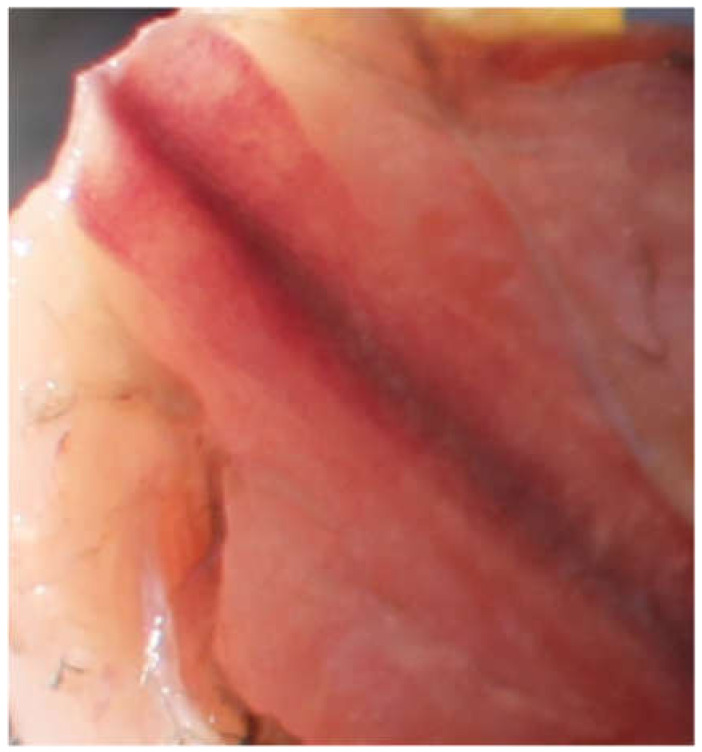
A normal keel bone tip with no fracture lines or callus.

Only one of the 29 keel bones presented a visible fracture during autopsy, see Table 1. The keel bone belonged to a female jungle fowl. The fracture was not palpable due to barely any callus formation and it was only detected during autopsy. The fracture site was located caudally, on the tip of the bone. The fracture was on the dorsal side of the bone, which is not accessible by external palpation. It was visible as a dark line with a slight elevation, observed both visually and detectable with the fingertip. Figure 1 shows the keel bone with fracture, Figure 2 is a comparable keel bone with no fracture. It must be noted that the color of the bone varied between individuals, from dark red towards pinker colors.

### 3.3. Palpation of Fractures versus Autopsy

One of the birds was during palpation thought to have a fracture, however necropsy did not confirm the finding. The bird with an observed fracture during autopsy had a false negative palpation score.

## 4. Discussions

Several of the suggested risk factors for KBD are linked to modern selection for egg production. If selection for efficient egg production is an underlying cause, an important step towards more knowledge is to investigate keel bones of the modern laying hen’s ancestor, the red jungle fowl. To the authors’ knowledge, this is the first study to examine keel bones from the red jungle fowl. The results indicate that keel bone fractures were not common in red jungle fowls in this study group, neither in male nor female specimens. Very slight deviations were common in females. The number of animals in the study is limited, thus a broad generalization of the results is not possible. In addition, the birds were not purebred jungle fowls; they originated from two different zoo populations crossed to increase genetic variability [29]. Nevertheless, the novel findings of the present study emphasize the need to further investigate the proposed hypothesis in large scale studies.

One out of 12 hens and none of the 17 roosters of the red jungle fowls in the present study had a keel bone fracture. The low number of KBF observed is in contrast to the high prevalence, ranging from 30 to 97%, reported in modern laying hens in previous studies [2,3,4]. Several of the factors associated with keel bone fractures in previous studies are linked to egg production, e.g., number of eggs and onset of lay [20]. Therefore, it was no surprise that all the 17 roosters had normal keels without fractures. This finding is in accordance with the results of Fleming et al. [27], one of the few studies that previously have investigated keel bones of roosters. Very slight deviations were frequent in the hens: Ten out of 12 hens had a deviation, while only one out of 17 males had a deviation. The prevalence of deviations was higher than the 6–59% reported in commercial laying hen lines at 60–62 weeks [2,32]. Since the deviations in the red jungle hens were very slight, it could be speculated that these would not be reported in modern layers, since they often exhibit severely deformed keel bones [33]. Deviations may be linked to perch material; soft perches have been found to reduce the incidence of deviations [34]. The red jungle hens had access to perches made of metal, which creates more pressure on the keel versus softer materials. The deviations in the hens were located in the cranial middle part of the keel bone. This is not the same anatomical region as most keel bone fractures previously reported, which are most commonly described to affect the dorsal and caudal tip of the bone [18]. The differences in morphology and location support the theory that keel bone fractures and keel bone deviations are two separate conditions with different etiology. This emphasizes the importance of making the distinction and must be taken into account in studies regarding KBD. Evaluation of the keel bones were performed by an experienced poultry veterinarian, trained by the EU Cost Keel Bone Action’s training school. One of the birds had a false positive palpation score, and the bird with an observed fracture during autopsy had a false negative palpation score. Even though the sample size was very limited, the findings support that palpation as the sole assessment method was hampered with inaccuracy [1,5,15,18,19]. The observed fracture was located dorsally at the caudal tip of the keel bone and was thus inaccessible for palpation.

The keel bone in commercial layers is not fully ossified until week 35–40 [35], which means that the modern hen starts laying eggs 15–20 weeks before the keel bone is fully matured. The majority of the keel bone fractures in layers are located caudally on the tip of the bone [18], which is the last part to be ossified. It has been speculated that the ossification zones are weak spots, prone to fractures [20,36]. The ossification of the red jungle fowl’s keel has never been investigated; therefore, we do not know if the late ossification of the caudal keel bone applies for both modern layer lines and red jungle fowl. Lack of knowledge regarding the ossification of the red jungle hen’s keel bone warrants further studies.

Several production parameters are different in the red jungle fowl compared to commercial, modern layers. Disentangling the effect of different production parameters warrants further investigation in future studies. The jungle hens in the current study were semi-selected; they had a relatively high production compared to non-selected fowls in the wild, with four to six eggs per breeding season [25]. Even though the production in the study group was high compared to wild jungle fowls, it is considerably lower than modern laying hen strains. Selection for increased egg efficiency in commercial hybrids has resulted in a peak production of 93–95% and up to 310 eggs laid annually [37,38]. A link between egg production and keel bone fractures have previously been documented by Eusemann et al. [39]. They treated hens with deslorelin acetate implant before the onset of laying, which leads to a suppression of follicles and, hence, no egg production. Treated hens had normal keel bones with no fractures. However, it has not been investigated if the hormonal status per se is associated with keel bone fractures. Another important production parameter in commercial layers is onset of lay. For the red jungle hens in this study, onset of lay was 23 weeks. Modern layers begin to lay eggs around 16–19 weeks of age [38]. A study by Gebhardt–Henrich and Fröhlich [4] found a positive association between early onset of lay and keel bone fractures observed end of lay. Egg production is also measured by the size and mass of the egg. The average egg weight for the red jungle hens in the current study was 30 g, with a body weight of 800 g. This is nearly half the egg weight compared to the modern layer, who has an average egg weight of around 62 g, with a body weight of 1300 g [38]. Keel bone fractures have been shown to affect production parameters, including an increase in egg weight [40,41]. However, the opposite effect, meaning that the size and weight of the egg may influence the prevalence of KBF, has not previously been investigated. This should be explored further in different hybrids and layer lines.

## 5. Conclusions

In conclusion, this pilot study found a very low prevalence of keel bone fractures and a high prevalence of keel bone deviations observed in a study group of 112-week-old red jungle fowls housed in aviary systems. The prevalence of fractures was low compared to previously published studies in modern layer hybrids, while the prevalence of deviations was high. It must be noted that the deviations were very slight. The present study was descriptive and thus the design did not allow for inferences on causality. In order to disentangle the effect of different factors on KBF, and to investigate the hypothesis that selective breeding might have contributed to an increase in keel bone pathology, large scale studies are needed.

## Figures and Tables

**Table 1:** Overview of keel bone findings in the jungle fowl.

	Hens ^1^			Roosters ^2^		
	*n*	%	95% CI ^3^	*n*	%	95% CI
**Fractures**						
Yes	1	8.3%	0–48%	0	0%	-
No	11	92%	52–99%	17	100%	-
**Deviations** ^4^						
Yes	10 ^3^	83%	47–97%	1 ^3^	5.9%	0.7–36%
No	2	17%	3.5–52%	16	94%	64–100%

^1^*n* = 12; ^2^ *n* = 17; ^3^ Confidence interval; ^4^ all observed deviations were very slight.
